# Is Preoperative Anticoagulation in Nephrectomy with Caval Thrombectomy Necessary? A Multicenter Retrospective Cohort Study

**DOI:** 10.1016/j.euros.2025.08.002

**Published:** 2025-08-26

**Authors:** Théophile Bertail, Zine-Eddine Khene, Raphaël Fleury, Thibault Waeckel, Louis Surlemont, Franck Bruyère, Nicolas Doumerc, Pierre Bigot, Morgan Rouprêt, Jean-Christophe Bernhard, Karim Bensalah

**Affiliations:** a Department of Urology, CHU Rennes, Rennes, France; b Department of Urology, CHU Caen Normandie, Caen, France; c Department of Urology, CHU Rouen, Rouen, France; d Department of Urology, CHRU Tours, Tours, France; e Department of Urology, CHU Rangueil, Toulouse, France; f Department of Urology, CHU Angers, Angers, France; g Urology, GRC 5 Predictive Onco-Uro, AP-HP, Pitie-Salpetriere Hospital, Sorbonne University, Paris, France; h Department of Urology, CHU Bordeaux, Bordeaux, France

**Keywords:** Kidney cancer, Thrombus, Renal cell carcinoma, Anticoagulation, Complications

## Abstract

**Background and objective:**

Surgery of renal cell cancer (RCC) with a caval thrombus (CT) is associated with significant morbidity, particularly regarding thromboembolic complications. There are no data or recommendations regarding the potential benefit of preoperative anticoagulants. We aimed to assess the usefulness of preoperative anticoagulation regarding surgical outcomes and thromboembolic events in patients undergoing nephrectomy with inferior vena cava thrombectomy.

**Methods:**

We conducted a multicenter retrospective study of 216 patients who underwent surgery for RCC with a CT. Of these patients, 114 had preoperative anticoagulants. Intraoperative data and postoperative complications, particularly thromboembolic events, were compared between the two groups. To account for potential selection biases arising from nonrandom allocation of patients to different groups, we performed a propensity score–matched analysis.

**Key findings and limitations:**

There were more overall and major complications in the anticoagulation group, but the difference became nonsignificant after matching (odds ratio [OR] 1.58; 95% confidence interval or CI [0.76–3.26]; *p* = 0.17 and OR 1.83; 95% CI [0.68–4.96]; *p* = 0.21 respectively). Other pre- and postoperative parameters, particularly thromboembolic events, did not differ between the two groups. The main limitations are the retrospective design and the intercenter variability.

**Conclusions and clinical implications:**

The prescription of anticoagulation at the time of diagnosis of RCC with a CT does not appear to have any influence on the occurrence of thromboembolic events, while potentially increasing morbidity.

**Patient summary:**

Surgery for kidney tumors with a caval thrombus is difficult and associated with a high rate of complications. One of the most feared complications is pulmonary embolism. Our study suggests that prescribing anticoagulants before surgery does not decrease the risk of embolism and could be responsible for an increased rate of complications.

## Take Home Message

Our study suggests that prescribing anticoagulants before surgery for kidney tumors with a caval thrombus does not decrease the risk of embolism and could be responsible for an increased rate of complications.

## 1 Introduction

Renal cell cancer (RCC) with a caval thrombus (CT) involving the inferior vena cava (IVC) represents approximately 4% of newly diagnosed patients [1,2]. When there is no metastasis, surgical removal of the tumor and thrombus is the recommended treatment [[3], [4], [5]]. However, this procedure is associated with significant morbidity: intra- and postoperative complications occur in up to 60% of cases [6,7]. Thromboembolic events, mostly pulmonary embolism, are among the most feared complications as these can sometimes be fatal [[8], [9], [10]].

The literature concerning the management of RCC with a thrombus is limited. Most of the publications are small retrospective studies originating from single centers. Many of these were published a long time ago. In a systematic review performed in 2016 by the European Kidney Cancer Guideline Group, only 14 articles were considered for final evaluation [11] and the authors underlined the high risks of bias and confounding. Most of the articles focus on oncological outcomes and surgical morbidity. There are no data regarding the usefulness/harms of perioperative anticoagulation (ATC). Given the high heterogeneity among urologists, we felt that clarifying this specific issue was of interest.

International guidelines recommend thromboprophylaxis after major cancer surgery such as nephrectomy and thrombectomy [12,13]. However, there is no recommendation regarding preoperative ATC at the time of diagnosis of RCC with a CT. In France, a recent survey among the urological community showed a wide variety of practices: a majority of surgeons (60%) prescribe anticoagulants (at a prophylactic or curative dose) while others do not (unpublished data). Some base their prescription of anticoagulants on the aspect of the CT (clotted vs tumoral). However, all practitioners who were surveyed admitted that their practice was not based on any evidence.

To clarify this issue, we evaluated the usefulness of preoperative ATC for surgical outcomes of patients undergoing nephrectomy with IVC thrombectomy.

## 2 Patients and methods

### 2.1 Study design and participants

We surveyed the French UroCCR database (UroCCR project NCT03293563), which is approved by the institutional review board and has received approval from Commission Nationale de l’informatique et des Libertés (number DR-2013-206). We performed a retrospective analysis of all patients from 18 French centers who underwent surgical resection of nonmetastatic RCC with a CT between January 2013 and July 2023. Patients with missing data of interest in the database were excluded. All the patients received oral and written information about the objectives and methodology of the UroCCR project, and written consent was obtained.

### 2.2 Covariates

Demographic, tumor, and pathological characteristics at the time of surgery were collected. Demographics included age, sex, Eastern Cooperative Oncology Group performance status, American Society of Anesthesiologists (ASA) classification, and anticoagulant/antiplatelet treatments. Tumor characteristics included tumor size, tumor side, thrombus level as described by Neves and Zincke [14], and nuclear grade.

### 2.3 Outcomes

Perioperative variables included operative time, estimated blood loss, intraoperative blood transfusion, and intraoperative complication rate. Postoperative variables included overall complication rate, major complication rate, blood transfusion rate, length of stay, postoperative thromboembolic events, and occurrence of hemorrhagic complications (defined as bleeding requiring reoperation or hematoma requiring transfusion). Postoperative complications were graded according to the Clavien-Dindo classification [15]. Major complications were defined as a Clavien score of ≥3. Postoperative thromboembolic events were defined as deep vein thrombosis or pulmonary embolism occurring between the time of diagnosis and 30 d postoperatively. Cancer-specific survival (CSS) was defined as the time from the date of diagnosis to the date of death from RCC, and overall survival (OS) was defined as the length of time from the date of diagnosis to the date of evaluation if patients diagnosed with RCC are still alive.

### 2.4 Statistical analysis

Continuous variables were expressed as medians with interquartile ranges, while categorical variables were presented as absolute numbers and percentages. To address the potential selection bias due to the nonrandom allocation of patients to treatment groups, a propensity score–matched analysis was performed. The propensity score, defined as the probability of receiving preoperative ATC conditional on baseline characteristics, was estimated using a multivariable logistic regression model that included age (as a continuous variable), ASA classification, and level of thrombus (both as categorical variables). Patients in the ATC group were matched 1:1 without replacement to patients in the nonanticoagulated (NATC) group using nearest-neighbor matching within a caliper width equal to 0.2 times the standard deviation of the logit of the propensity score, in accordance with the standard recommendations.

After matching, all statistical analyses accounted for the matched-pair design. Continuous outcomes were compared using linear regression models with robust standard errors clustered by matched pairs. Binary outcomes were analyzed using conditional logistic regression to appropriately account for the dependency within matched pairs. This approach provided estimates of treatment effect in the form of odds ratios (ORs) with corresponding 95% confidence intervals (CIs). For descriptive purposes, unmatched comparisons were performed using the Mann-Whitney U test for continuous variables and chi-square tests for categorical variables. OS and CSS were assessed using Kaplan-Meier estimates, with group comparisons using the log-rank test. Additionally, we performed Cox proportional hazard regression models adjusted for matching using robust variance estimators to report hazard ratios (HRs) and 95% CIs. All statistical tests were two sided, with a significance level set at *p* < 0.05. Analyses were conducted using Stata version 14.1 (StataCorp LP, College Station, TX, USA).

## 3 Results

Baseline characteristics are presented in Table 1. A total of 216 patients were analyzed, of whom 114 received preoperative ATC. Within the ATC group, 92 patients received prophylactic ATC, while 19 had curative ATC. Of the patients, 20% in the ATC group were already treated by ATC (for a reason unrelated to cancer) before an RCC diagnosis. There were small differences between the two groups. The ATC group had a higher proportion of ASA 3–4 patients than the NATC group (59% vs 30%). Additionally, ATC patients had a higher CT level (64% of level III–IV CT vs 32% in the NATC group). There was a majority of clear cell carcinomas in both groups (84% in the ATC group and 89% in the NATC group).

**Table 1:** Patients’ characteristics—all patients and after propensity score matching

	Full data set			PS-matched patients		
	No preoperative anticoagulant (*n* = 102)	Preoperative anticoagulant (*n* = 114)	SMD	No preoperative anticoagulant (*n* = 64)	Preoperative anticoagulant (*n* = 64)	SMD
Age (yr), median (Q1, Q3)	68.24 (61.13, 76.02)	69.77 (60.41, 76.17)	0.135	68.24 (60.23, 75.45)	69.33 (58.56, 75.81)	0.09
Gender—male, *n* (%)	72 (70.59)	84 (73.68)	0.069	45 (70.31)	50 (78.12)	0.129
ASA classification, *n* (%)						
1–2	65 (69.89)	42 (40.38)	0.593	36 (56.25)	41 (64.06)	0.105
3–4	28 (30.11)	62 (59.62)		28 (43.75)	23 (35.94)	
History of TED, *n* (%)	3 (2.94)	27 (24.11)	0.619	33 (56.90)	25 (42.37)	0.120
Initiation, *n* (%)						
De novo		89 (41.59)			13 (10.32)	
Chronic		23 (10.75)			49 (38.89)	
Type of anticoagulant, *n* (%)						
Preventive		92 (82.88)			49 (80.33)	
Curative		19 (17.12)			12 (19.67)	
ECOG performance status ≥1, *n* (%)	32 (33.68)	28 (32.94)	0.016	19 (35.85)	14 (25.45)	0.096
Laterality—right, *n* (%)	68 (66.67)	73 (64.04)	0.055	39 (60.94)	37 (57.81)	0.064
Tumor size (cm), median (Q1, Q3)	9 (7, 11)	8.75 (6.5, 11)	0.079	9.5 (6.5, 12)	9 (6, 11.3)	0.095
Thrombus height, *n* (%)						
IVC <2 cm	51 (50.00)	37 (32.74)	0.350	28 (43.75)	33 (51.56)	0.106
IVC >2 cm	31 (30.39)	43 (37.71)		22 (34.38)	18 (28.12)	
IVC above hepatic veins below diaphragm	28 (27.45)	62 (54.38)		9 (14.06)	9 (14.06)	
IVC above diaphragm	5 (4.90)	11 (9.64)		5 (7.81)	4 (6.25)	
Histology, *n* (%)						
ccRCC	91 (89.22)	96 (84.96)	0.127	58 (90.62)	52 (82.54)	0.117
Others	11 (10.78)	17 (15.04)		6 (9.38)	11 (17.46)	
Nuclear grade, *n* (%)						
1–2	83 (93.26)	6 (6.74)	0.325	2 (3.57)	5 (8.62)	0.120
3–4	92 (87.62)	13 (12.38)		54 (96.43)	53 (91.38)	
Stage, *n* (%)						
0	47 (46.53)	47 (42.34)	0.084	34 (53.12)	29 (47.54)	0.102
1	14 (13.86)	13 (11.71)		10 (15.62)	7 (11.48)	
2	40 (39.60)	51 (45.95)		20 (31.25)	25 (40.98)	

ASA = American Society of Anesthesiologists; ccRCC = clear cell renal cell cancer; ECOG = Eastern Cooperative Oncology Group; IVC = inferior vena cava; PS = propensity score; SMD = standardized mean difference.

Using 1:1 propensity score matching considering age, ASA score, and the level of thrombus, we obtained two groups of 64 ATC/NATC patients with comparable characteristics (Table 2). The operative time was similar in both groups (mean difference [MD] 4.63; 95% CI [–39.43 to 30.17]; *p* = 0.9). There was no difference regarding estimated blood loss before or after matching (MD 337; 95% CI [–882 to 208]; *p* = 0.135). There was a higher incidence of overall complications in the ATC group (41.2% vs 22%; *p* = 0.003) before matching, but the difference became nonsignificant after matching (OR 1.58; 95% CI [0.76–3.26]; *p* = 0.17). The major complications were more common in the ATC group before matching (22.8% vs 9.8%, *p* = 0.01), but similar after matching (OR 1.83; 95% CI [0.68–4.96]; *p* = 0.21). Hemorrhagic complications, postoperative transfusion, need for reintervention, and postoperative thromboembolic events were not different between the groups before and after matching. The length of stay was comparable between the groups (7 vs 7 d; MD 2.45; 95% CI [–6.19 to 1.30]; *p* > 0.9).

**Table 2:** Peri- and postoperative outcomes—all patients and after propensity score matching

Outcome	Before propensity score matching			After propensity score matching				
	No ATC (*n* = 102)	ATC (*n* = 114)	*p* value	No ATC (*n* = 64)	ATC (*n* = 64)	MD/OR	95% CI	*p* value
Operative time (min), median (IQR)	210 (150–246.5)	210 (180–255)	0.54	211 (150–255)	205 (180–249)	–4.63	(–39.43 to 30.17)	0.9
EBL (ml), median (IQR)	1200 (475–2000)	1000 (550–2000)	0.76	1500 (750–2100)	1000 (500–2000)	–337.16	(–882.63 to 208.32)	0.135
Intraoperative complications, *n* (%)	19 (19.00)	34 (29.82)	0.07	13 (20.63)	17 (26.56)	1.31	(0.63–2.72)	0.43
Overall complications, *n* (%)	23 (22.55)	47 (41.23)	0.003	15 (23.44)	22 (34.38)	1.58	(0.76–3.26)	0.17
Major complications, *n* (%)	10 (9.80)	26 (22.81)	0.01	7 (10.94)	12 (18.75)	1.83	(0.68–4.96)	0.21
Hemorrhagic complications, *n* (%)	8 (8.00)	16 (14.16)	0.16	5 (8.06)	5 (7.94)	1.00	(0.25–3.99)	>0.9
Length of stay (d), median (IQR)	7.5 (5–14)	7 (6–11)	0.74	7 (5–13)	7 (6–12.5)	–2.45	(–6.19 to 1.30)	>0.9
Postoperative transfusion, *n* (%)	17 (17.00)	27 (23.68)	0.23	12 (19.35)	12 (18.75)	0.45	(0.20–0.99)	>0.9
Postoperative TE event, *n* (%)	16 (16.00)	20 (17.70)	0.74	11 (17.74)	12 (19.05)	1.11	(0.45–2.75)	0.85
Reintervention, *n* (%)	7 (7.00)	15 (13.16)	0.14	5 (8.06)	5 (7.81)	1.00	(0.25–3.99)	>0.9

ATC = anticoagulant group; CI = confidence interval; EBL = estimated blood loss; IQR = interquartile range; MD = mean difference; No ATC = nonanticoagulant group; OR = odds ratio; TE = thromboembolism.

Continuous variables were compared using linear regression models with robust standard errors clustered by matched pair. Binary outcomes were analyzed using conditional logistic regression to account for the matched-pair design. Odds ratios and 95% confidence intervals are reported for binary outcomes. Median and IQR are shown for descriptive purposes. A *p* value of <0.05 was considered statistically significant.

**Fig. 1:**
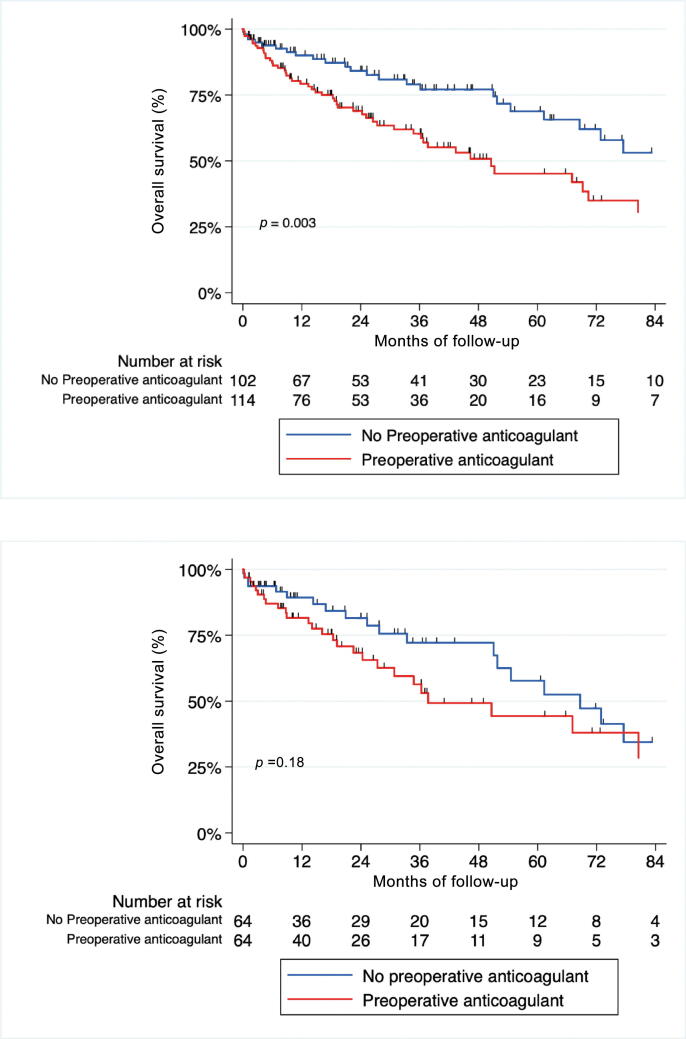
Kaplan-Meier curves estimates of overall survival for patients with nonmetastatic renal cell carcinoma and IVC-TT treated with surgery (A) before and (B) after propensity score matching. IVC-TT = inferior vena cava thrombectomy.

**Fig. 2:**
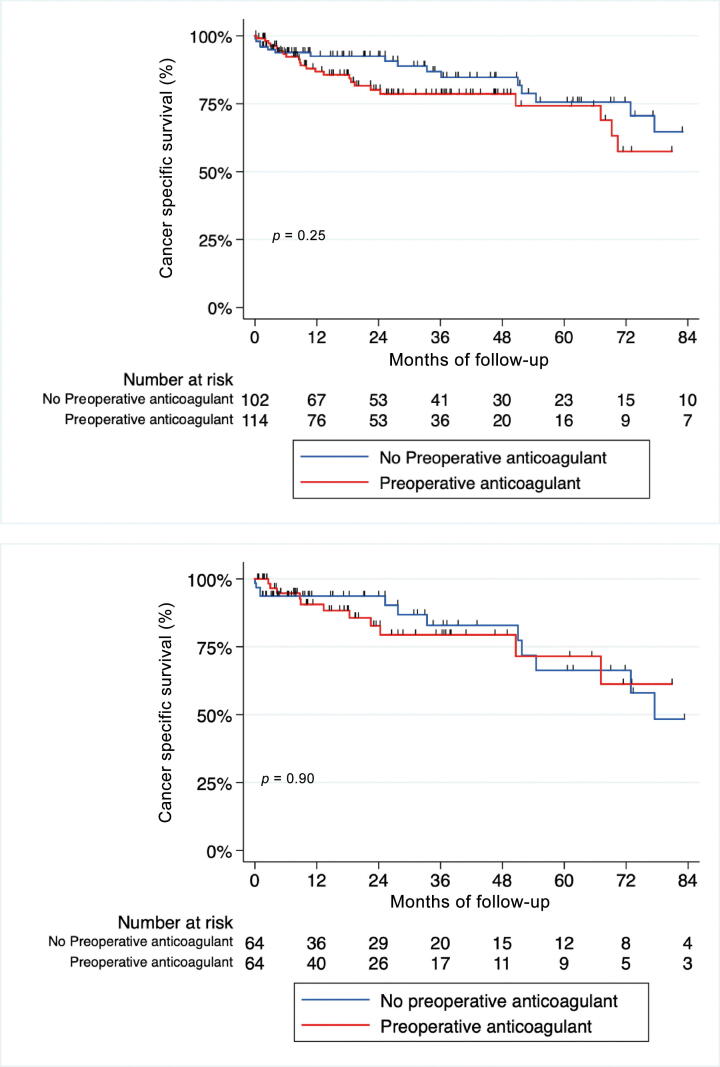
Kaplan-Meier curves estimates of cancer-specific survival for patients with nonmetastatic renal cell carcinoma and IVC-TT treated with surgery (A) before and (B) after propensity score matching. IVC-TT = inferior vena cava thrombectomy.

After a median follow-up of 32 mo, 77 patients died, including 42 from RCC. The median follow-up of patients without any event (censored) was 26.15 mo**.** Unadjusted Kaplan-Meier curves for CSS and OS according to ATC are shown in Figures 1A and 2A. Median OS was shorter in the ATC groups (HR = 2.02; 95% CI [1.25–3.27]; *p* < 0.05; Fig. 1A), but there was no difference regarding cancer-related death (HR = 1.44; 95% CI [0.76–2.71]; *p* = 0.25; Fig. 2A). In the matched cohort, there was no difference in OS or CSS between the two groups (HR = 1.5; 95% CI [0.83–2.72]; *p* = 0.18, and HR = 1.05; 95% CI [0.46–2.4]; *p* = 0.9, respectively; Fig. 1B and 2B).

## 4 Discussion

Nephrectomy associated with IVC thrombectomy carries an important risk of perioperative complications, with reported morbidity rate being as high as 70% and mortality rates ranging from 2% to 10% [16,17]. The most common complication is major blood loss [16], particularly in patients with a high level of CT [17] and/or requiring cardiopulmonary bypass [18].

The occurrence of thromboembolic events in the perioperative period is a major concern since it can increase morbidity and lead to patient death in case of massive embolism. For this reason, many physicians involved in the care of these patients (urologists, oncologists, and anesthesiologists) willingly put patients on anticoagulants when a CT is diagnosed, probably hoping that it will prevent thromboembolic complications. However, there are no data regarding the clinical impact of ATC on the risk of venous thromboembolism (VTE) in RCC patients with a CT. Although the issue is often debated, there is no evidence or guidelines to recommend the use of ATC at the time of diagnosis in these patients while waiting for surgery.

We were unable to detect a difference between the two groups regarding the occurrence of VTE before or after surgery. Although the scientific literature on the subject is very limited, our findings are in line with the publications on other solid tumors. A pediatric study of solid tumors with tumor thrombus showed no difference in VTE and thrombus formation with or without ATC, but reported increased bleeding in anticoagulated patients [19]. A retrospective study of patients with both RCC and hepatocellular carcinoma associated with tumor thrombus found that VTE and hemorrhagic complications were more common in patients receiving ATC [20]. This limited evidence suggests that it is not necessary to prescribe ATC in patients with a CT.

We also observed that ATC was associated with increased morbidity: patients in the ATC group had more overall (41% vs 22%) and major (23% vs 10%) complications, which can be seen as another incentive to avoid ATC. However, patients in the ATC population had more comorbidities and more complex CTs. Hence, differences disappeared once the populations were propensity matched.

There are some limitations that must be considered when interpreting our results. First, as mentioned already, this study is limited by its retrospective design, resulting in a selection bias. ATC and NATC groups had some differences: ATC patients had more comorbidities (twice as many ASA 3–4 patients as those in the NATC group) and higher CT levels (again twice as many stage III–IV CT patients as those in the NATC group). Moreover, a significant proportion of patients (20%) were already receiving ATC because of specific morbidities such as atrial fibrillation or prosthetic cardiac valve. Our database does not show the details of comorbidities. However, it is very likely that the anticoagulant treatment can be seen as a marker of either increased comorbidities, or larger tumors and/or thrombus, which explains that before matching, there were more complications in the ATC group. Second, large multicenter databases can include poorly classified or reported variables that can affect the quality of any analysis. Finally, many surgical teams were involved, which might have biased our results. There are limited data on the specific morbidity of surgery for renal tumors with a CT. There are big series that report up to 10% mortality and 40% complications [11,21]. Although every surgeon knows that it is a complex and morbid procedure, there are no data regarding the impact of surgeon/center volume on the perioperative outcomes of caval thrombectomy. However, it is very likely that intercenter variability has influenced our results. There are many differences across centers regarding surgeon’s experience, collaboration with practitioners of other specialties (hepatic surgeons, vascular surgeons, cardiac surgeons, and anesthesiologists), and personal beliefs regarding the usefulness of anticoagulant treatment. This heterogeneity is difficult to evaluate, but has probably influenced the outcomes and will always make it difficult to set ambitious prospective studies on kidney cancer with CT. Finally, it is well known that propensity score matching results in a loss of power and precision of estimates due to sample size loss.

However, we believe that our results are interesting because the literature on the subject is very sparse. This is the first multicenter and the highest-powered study to specifically focus on the subject. To mitigate confounding arising from the absence of randomization, we used propensity score pair matching to balance observed the baseline characteristics between groups. This method can improve comparability by reducing the bias due to measured confounders. However, it does not guarantee complete balance and cannot address unmeasured confounding. Of course, a randomized trial would be the best way to address this issue. We considered conducting a prospective randomized study, but our methodology evaluation showed that it would require too much time and resources: around 500 patients should be recruited, which makes it highly unlikely to happen. Our study, although retrospective, provides the first elements of an answer.

## 5 Conclusions

Prescribing ATC at the time of diagnosis of kidney cancer with IVC thrombus does not decrease the occurrence of VTE and could be associated with increased morbidity. Although limited by the biases inherent to retrospective collection of data, these results suggest that preoperative ATC at the diagnosis of RCC with a CT is not necessary.

  ***Author contributions*:** Karim Bensalah had full access to all the data in the study and takes responsibility for the integrity of the data and the accuracy of the data analysis.

  *Study concept and design*: Bertail, Khene, Bensalah.

*Acquisition of data*: Fleury, Waeckel, Surlemont, Bruyère, Doumerc, Bigot, Rouprêt, Bernhard.

*Analysis and interpretation of data*: Bertail, Khene, Bensalah.

*Drafting of the manuscript*: Bertail, Khene, Bensalah.

*Critical revision of the manuscript for important intellectual content*: Bertail, Khene, Bensalah.

*Statistical analysis*: Khene.

*Obtaining funding*: None.

*Administrative, technical, or material support*: None.

*Supervision*: Bensalah.

*Other*: None.

  ***Financial disclosures:*** Karim Bensalah certifies that all conflicts of interest, including specific financial interests and relationships and affiliations relevant to the subject matter or materials discussed in the manuscript (eg, employment/affiliation, grants or funding, consultancies, honoraria, stock ownership or options, expert testimony, royalties, or patents filed, received, or pending), are the following: None.

  ***Funding/Support and role of the sponsor*:** None.

  ***Acknowledgments*:** The authors acknowledge the following coauthors: Jonathan Olivier, Fayek Taha, Nicolas Branger, Constance Michel, Hervé Lang, Maxime Vallee, Jean-Jacques Patard, Clément Sarrazin, Romain Boissier, and Cécile Champy.
